# Navigating the potassium dilemma: a qualitative study of nephrologists’ strategies for renin–angiotensin–aldosterone system inhibitor preservation and hyperkalaemia management in Spain

**DOI:** 10.1371/journal.pone.0354854

**Published:** 2026-07-30

**Authors:** Nicolas Gimenez-Stamminger, Ceu Mateus, Thomas Mason, Andrew Harding

**Affiliations:** Faculty of Health and Medicine, Division of Health Research, Lancaster University, Lancaster, United Kingdom; Stellenbosch University Faculty of Medicine and Health Sciences, SOUTH AFRICA

## Abstract

**Background:**

Hyperkalaemia is a common complication among patients with predialysis chronic kidney disease (CKD) and a leading reason for modifying or discontinuing renin–angiotensin–aldosterone system inhibitor (RAASi) therapy despite its proven cardiorenal benefits. Although newer potassium binders may improve potassium control while enabling RAASi use, real clinical practice remains poorly characterised. This study explored how nephrologists in Spain manage hyperkalaemia in patients with predialysis CKD (stages 3–5), with particular emphasis on RAASi continuation.

**Methods:**

Semi-structured interviews were conducted with 12 practising nephrologists across Spain who routinely manage hyperkalaemia in patients with predialysis CKD. Interviews were transcribed verbatim and analysed using ATLAS.ti. A codebook-based thematic analysis was performed, with codes iteratively refined into 15 sub-themes and grouped into five overarching themes.

**Results:**

Five themes were identified: (1) hyperkalaemia was managed using a structured clinical algorithm incorporating dietary, metabolic, diuretic, and pharmacological strategies, to preserve RAASi therapy where possible; (2) newer potassium binders (patiromer and sodium zirconium cyclosilicate) were viewed as effective and better tolerated than traditional resins, supporting greater RAASi continuity; (3) system-level barriers, including *visado* requirements, regional formulary variation, and fragmented communication across specialties, limited timely use of newer binders were perceived to contribute to RAASi discontinuation; (4) patient engagement, underpinned by clear communication, trust, and nursing-led education, facilitated adherence, although clinician-level nutrition expertise varied; and (5) hyperkalaemia management affected patients’ daily functioning and emotional well-being, yet formal assessment of health-related quality of life (HRQoL) was infrequent.

**Conclusions:**

Nephrologists in Spain prioritised preservation of RAASi therapy when managing hyperkalaemia in predialysis CKD. Although newer potassium binders supported this objective, their use was limited by access and care coordination challenges. Streamlined access pathways, improved multidisciplinary communication, and routine HRQoL assessments may enable more patient-centred and guideline-concordant care. These implications reflect qualitative clinician perspectives rather than evidence of clinical or economic effectiveness.

## Introduction

Hyperkalaemia is a common and potentially life-threatening electrolyte disturbance in patients with predialysis chronic kidney disease (CKD; stages 3–5) [1]. Impaired renal potassium excretion, together with comorbidities such as heart failure and diabetes and the widespread use of renin–angiotensin–aldosterone system inhibitors (RAASis), increases both the incidence of hyperkalaemia and the risk of adverse outcomes [2]. Observational studies indicate that elevated serum potassium concentrations (commonly defined as ≥5.1 mmol/L) are associated with higher mortality, cardiovascular events, and renal disease progression [3,4].

This creates a clinical conflict, often referred to as the “potassium dilemma.” The cardiovascular and renal benefits of RAASi therapy, including angiotensin-converting enzyme inhibitors (ACEis), angiotensin receptor blockers (ARBs), and steroidal and non-steroidal mineralocorticoid receptor antagonists, are supported by high-quality evidence and recommended in clinical guidelines [5–8]. These guidelines also advise using the highest tolerated doses of RAASi for optimal therapeutic effect [9,10]. However, real-world data suggest that RAASi doses are frequently reduced or discontinued following the development of hyperkalaemia [11,12].

Discontinuing RAASi has been associated with increased mortality and adverse cardiorenal outcomes compared with continued maximal therapy [13–16]. Therefore, strategies that prevent hyperkalaemia and support continued optimal RAASi therapy are of therapeutic interest. Potassium binders, including sodium polystyrene sulfonate, calcium polystyrene sulfonate (CPS), and the more recently approved sodium zirconium cyclosilicate (SZC) and patiromer, effectively reduce excess potassium ions by binding them in the gastrointestinal tract [17]. CPS use has been limited by variable serum potassium-lowering effects and a higher incidence of gastrointestinal adverse events [18,19]. In contrast, patiromer and SZC have demonstrated improved tolerability and effectiveness in managing serum potassium [20–24]. Evidence suggests that patiromer enables continued RAASi therapy in patients with CKD, heart failure, and hypertension by reducing serum potassium [21,23,25–30], whereas SZC optimises RAASi in smaller patient cohorts [31]. Recent real-world data from the United States, Japan, and Spain indicate that longer outpatient treatment with SZC is associated with a higher likelihood of continued RAASi therapy, although the protective effect diminishes following SZC discontinuation [32].

The efficacy of these agents is established; however, their use in routine clinical practice remains poorly understood. This is particularly relevant in Spain, where the National Health System provides universal healthcare coverage, but access to newer potassium binders is subject to specific administrative controls, including *visado*, a mandatory prior authorisation process for selected medicines that may delay or restrict prescribing in routine practice [33]. Understanding how nephrologists experience and respond to these access conditions is important because administrative and organisational factors may influence whether evidence-based therapies can be implemented consistently in everyday care.

In this study, we explored nephrologists’ perspectives and clinical decision-making when managing hyperkalaemia among patients with predialysis CKD (stages 3–5) in Spain. Specifically, we examined how clinicians navigate the “potassium dilemma,” including their rationale for continuing or down-titrating RAASi, perceptions of potassium binders, and the influence of system-level constraints, patient-related factors, and perceived health-related quality-of-life (HRQoL) considerations. As this was a qualitative study, the objective was not to estimate treatment effects or test statistical associations, but to characterise clinicians’ experiences, reasoning, and perceived barriers in routine practice.

## Materials and methods

### Study design

We conducted a qualitative study using semi-structured interviews to explore nephrologists’ perspectives on the management of hyperkalaemia in patients with predialysis CKD (stages 3–5) in Spain. A qualitative approach was used to capture the clinical reasoning, perceived barriers to optimal treatment, and contextual factors influencing the use of RAASi and newer potassium binders. The study did not involve recruitment of patients or collection of patient-level clinical outcome data; instead, it focused on clinicians’ experiences and decision-making in routine practice. Accordingly, no inferential statistical analyses, hypothesis testing, confidence intervals, or multivariable models were planned or conducted. This study is reported in accordance with the Consolidated Criteria for Reporting Qualitative Research (COREQ) guidelines [34].

### Participants

Participants were eligible if they: 1) were practising nephrologists based in Spain; 2) were directly involved in the management of patients with CKD (stages 3–5); 3) had experience treating hyperkalaemia; 4) had ≥ 3 years of independent clinical practice; and 5) had documented experience prescribing traditional resins and at least one of the newer potassium binders (patiromer and/or SZC) within the previous 12 months. Nephrologists without regular direct patient-care responsibilities or prescribing experience in both binder classes (traditional and newer) were excluded. As participants were clinicians rather than patients, eligibility criteria were based on professional role, clinical experience, prescribing experience, and direct involvement in the management of predialysis CKD and hyperkalaemia.

Purposive sampling was used to include participants with varying years of clinical experience, practice settings, and geographical locations to capture diverse perspectives on regional health administration. Participants were recruited from six autonomous communities (Andalusia, Castilla-La Mancha, Castilla y León, Catalonia, Madrid, and Valencia), representing different regional health systems. The sample included clinicians from universities and general hospitals. Potential participants were identified through professional networks and invited via email. Interested clinicians received a participant information sheet outlining the study objectives, procedures, confidentiality, and terms of voluntary participation. Twelve nephrologists provided written informed consent and completed interviews. Recruitment ceased when the research team determined that data sufficiency had been reached, defined as the point at which no substantially new concepts emerged. The sample size was therefore justified on qualitative grounds, based on purposive sampling, diversity of participant characteristics, and the stability of emerging themes across interviews rather than statistical power. Participants’ demographics are summarised in **Table 1**.

**Table 1:** Characteristics of the participants (N = 12).

Demographic	Participant (N = 12),n (%)
**Years in clinical practice**	
**10–15 years**	3 (25)
**16–25 years**	7 (58)
**>25 years**	2 (17)
**Sex**	
**Male**	8 (67)
**Female**	4 (33)
**Primary practice setting**	
**University / Tertiary Hospital**	10 (83)
**General Hospital**	2 (17)
**Geographical Region (autonomous Community)**	
**Catalonia**	4 (33)
**Madrid**	3 (25)
**Andalusia**	2 (17)
**Castilla-la mancha**	1 (8)
**Castilla y león**	1 (8)
**Valencia**	1 (8)
**Prescribed new potassium binders (in the past 12 months)**	
**Veltassa (patiromer)**	6 (50)
**Lokelma (sodium zirconium cyclosilicate)**	6 (50)
**Age (years)**	
**40–49**	4 (33)
**50–59**	6 (50)
**≥60**	2 (17)

Note: Percentages may not total 100% owing to rounding. N, total number of participants; n, number of participants in the specific category.

### Data collection

A semi-structured interview guide was iteratively developed to elicit nephrologists’ experiences and decision-making processes when managing hyperkalaemia in patients with predialysis CKD (stages 3–5). The guide was informed by a review of the published literature and informal clinical discussions with practising nephrologists regarding the management of hyperkalaemia in predialysis CKD. We pilot-tested the guide with two nephrologists to assess the relevance and clarity of the interview questions and prompts. Following the pilot interviews, minor refinements were made, primarily involving wording adjustments to improve clarity and the addition of probes exploring access to newer potassium binders and multidisciplinary communication. Data from the pilot interviews were excluded from the analysis.

The final interview guide (**S1 File**) invited clinicians to reflect on: (1) their overall clinical approach to hyperkalaemia management; (2) the use and sequencing of dietary, pharmacological, and procedural interventions; (3) their experience prescribing traditional resins and newer potassium binders; (4) approaches to maintaining or adjusting RAASi therapy; (5) health-system or organisational factors influencing treatment decisions; and (6) patient-level considerations, including education, treatment adherence, and the perceived impact of hyperkalaemia management on HRQoL.

Demographic information (including years in practice, primary clinical setting, and geographical region) was collected via a brief electronic screener completed prior to the interview. Interviews were conducted in Spanish in October 2025 by a trained qualitative researcher via video conference. Each interview was held in a private setting, lasted approximately 45–60 min, and began with oral confirmation of informed consent. Interviews were digitally recorded and professionally transcribed verbatim. Transcripts were subsequently reviewed, cleaned, and pseudonymised by the research team prior to analysis. Analysis was conducted using the Spanish-language transcripts; illustrative quotations selected for publication were translated into English after analysis to preserve the meaning of participants’ accounts.

### Analysis

A thematic analysis following the six-phase approach described by Braun and Clarke [35], adapted for a structured, team-based codebook, was employed. This approach combined inductive coding grounded in the data with deductive coding informed by the study objectives and prior literature, and is commonly used in applied health research to enhance analytic transparency and consistency [36,37].

The transcripts were imported into ATLAS.ti version 25 (ATLAS.ti Scientific Software Development GmbH, Berlin, Germany). Two researchers independently coded an initial subset of transcripts to develop a draft codebook that combined inductive codes derived from the data and deductive codes reflecting predefined areas of interest (such as RAASi management and binder use). The initial coding subset was used to assess consistency in code interpretation and to refine code definitions, inclusion and exclusion criteria, and illustrative examples. Coding discrepancies were resolved through discussion, and the codebook was iteratively refined during analysis of subsequent transcripts in accordance with the familiarisation, coding, and refinement stages of thematic analysis. Once the codebook had been stabilised, the remaining transcripts were coded using the agreed coding framework, with additional team discussions held when new or ambiguous concepts emerged.

During the formal analysis phase, codes were compared within and across interviews to identify patterns and conceptual relationships. Through systematic comparison and team discussions, codes were grouped into higher-order categories and developed into overarching themes consistent with established approaches to thematic analysis in qualitative health research [38]. After code consolidation, 30 final codes were organised into 15 sub-themes and five overarching themes, reflecting clinical, system-level, and patient-centred aspects of hyperkalaemia management.

Rigour was supported through peer debriefing, maintenance of an audit trail documenting coding decisions and theme development, and regular reflexive discussions to minimise interpretive bias. The audit trail included successive versions of the codebook, notes on code merging and refinement, and documentation of decisions made during theme development. Reflexive discussions considered how the research team’s prior knowledge of CKD, hyperkalaemia management, and health-system access issues could influence interpretation of the data. Data sufficiency was assessed based on the stability of emerging concepts across interviews, with no substantively new themes identified in later transcripts [39].

### Ethical considerations

Data collection began after the research protocol was approved by the Ethics Committee of Lancaster University, United Kingdom (Approval no.: FHM-2025–4671-RECR-4). All participants received a participant information sheet before enrolment and provided written informed consent before taking part in the interview. Transcripts were pseudonymised, and data were stored on secure, access-restricted servers. The study was conducted in accordance with the principles of the Declaration of Helsinki.

### Data availability statement

The data underlying this study consist of qualitative interview transcripts from practising nephrologists in Spain. Full transcripts cannot be made publicly available because they contain potentially identifying contextual information, including participants’ clinical roles, regional practice settings, institutional contexts, and experiences with local access pathways for potassium binders. Given the small number of specialist participants and the specificity of the information provided, complete anonymisation cannot be guaranteed without substantially reducing the interpretive value of the data.

To support transparency and reproducibility, four Supporting Information files are provided: the semi-structured interview guide (**S1 File**), the codebook and code frequencies (**S2 Table**), anonymised participant characteristics (**S3 Table**), and anonymised illustrative quotations organised by theme (**S4 Table**). Requests for access to additional de-identified qualitative excerpts may be directed to the corresponding author. Requests will be reviewed in consultation with the research supervisor and, where necessary, the Ethics Committee of Lancaster University, United Kingdom. Access will be considered subject to applicable ethical, legal, and confidentiality requirements, including the terms of participant consent and the approval granted by the Ethics Committee of Lancaster University, United Kingdom.

## Results

Twelve nephrologists from across Spain participated in the study. All practised in hospital-based settings, including university, tertiary, and general hospitals. Three-quarters (75%) of participants had more than 15 years of clinical experience. All were actively involved in treatment decision-making for patients with predialysis CKD (stages 3–5) and recurrent hyperkalaemia. All participants had recent experience prescribing newer potassium binders, with usage in the past year evenly distributed between patiromer (50%) and SZC (50%).

Nephrologist interviews averaged 53 min (range: 45–60 min). A total of 30 codes were identified, with their frequencies and distributions provided in S1 Table. Five key themes and 15 subthemes characterising the real-world management of hyperkalaemia in patients with predialysis CKD emerged from the analysis, as summarised in Table 2.

**Table 2:** Study themes and sub-themes.

Main themes	Sub-themes
Safeguarding cardiorenal protection through stepwise clinical reasoning	• Staged management beginning with dietary and metabolic correction • Maintaining renin–angiotensin–aldosterone system inhibitors as a primary clinical imperative • Shifting toward individualised and evidence-based dietary practice
A therapeutic shift: new binders as enablers of care	• Modern binders viewed as a fundamental shift in outpatient management • Superior tolerability and usability compared with traditional resins • Facilitating the continuity of life-saving therapies in predialysis chronic kidney disease
Navigating systemic friction and care fragmentation	• Navigating inconsistent access and administrative hurdles to treatment • Overcoming fragmented coordination across medical specialties • Addressing the slow adoption of remote monitoring and digital innovations
The human element: trust, communication, and the role of nursing	• Fostering treatment adherence through transparent clinician communication • The essential role of renal nursing in patient education and empowerment • Identifying structural constraints in the delivery of comprehensive nutritional guidance
The unmeasured burden: when biochemistry overshadows lived experience	• Understanding how hyperkalaemia management restricts social and daily participation • Recognising the restoration of well-being through improved pharmacological tolerance • The challenge of relying on clinical intuition over formal quality-of-life assessment

### Safeguarding cardiorenal protection through stepwise clinical reasoning

Nephrologists consistently described hyperkalaemia as an expected and recurrent feature of advanced CKD, rather than an isolated biochemical abnormality. Their accounts reflected a shared clinical logic in which potassium management was embedded within a broader strategy to preserve long-term cardiorenal protection. Rather than reacting to isolated potassium values, clinicians emphasised a structured, stepwise approach aimed at maintaining stability while minimising disruption to disease-modifying therapies.

Initial management typically focused on conservative measures, including dietary counselling, optimisation of diuretic therapy, and correction of metabolic acidosis. These interventions were considered essential, particularly in patients with residual renal function, although clinicians acknowledged their limited effectiveness in advanced disease stages. As one participant explained, management followed a sequential logic:

*“I structure the management in a stepped way: first line, diet and diuretics; second line, bicarbonate or binders; third line, reduction or suspension of drugs that increase potassium, as a last resort.”* (ID05, Female, 56 years)

Central to this reasoning was the preservation of RAASis, which were considered non-negotiable owing to their established renal and cardiovascular benefits. Dose reduction or discontinuation was framed as a measure of last resort, considered only when potassium control could not be achieved through other strategies:

*“If we reduce or stop ACEis or ARBs, we may lower potassium, but we are also removing nephro- and cardioprotective drugs. In the medium and long term, that increases morbidity and mortality.”* (ID01, Male, 55 years)

Alongside this pharmacological reasoning, nephrologists described a gradual shift away from rigid dietary restrictions towards more individualised and evidence-based counselling. Participants critically reflected on earlier practices based on blanket restrictions of fruits and vegetables, noting poor adherence and unintended nutritional consequences:

*“Traditionally, there was a lot of insistence on restricting fruits and vegetables… but now we know that being too strict can be counterproductive and deprive patients of important nutrients.”* (ID06, Male, 51 years)

Together, these accounts illustrate how clinicians operationalised guideline principles in everyday practice, balancing biochemical control with the long-term imperative to safeguard cardiorenal protection.

### A therapeutic shift: new binders as enablers of care

The introduction of newer potassium binders was widely described as a turning point in hyperkalaemia management. Nephrologists characterised these agents as fundamentally changing what could be achieved in outpatient care, particularly for patients in whom hyperkalaemia previously led to recurrent emergency visits or withdrawal of essential therapies.

Participants contrasted newer binders with traditional resins, which were acknowledged to be pharmacologically effective but poorly tolerated. Gastrointestinal side effects, unpleasant texture, and taste were described as major barriers to sustained use, often resulting in poor adherence:

*“Resincalcio works, but many patients do not take it. They collect it from the pharmacy, but it never passes their mouth.”* (ID01, Male, 55 years)

In contrast, newer binders, such as patiromer and SZC were considered more predictable, better tolerated, and easier to integrate into daily life. Clinicians linked improved tolerability directly to better adherence and greater therapeutic continuity:

*“The arrival of the new binders has been a revolution. I have not had to dialyse patients urgently for hyperkalaemia for years, thanks to these treatments.”* (ID09, Male, 64 years)

Crucially, these agents were framed not merely as potassium-lowering drugs, but as enablers of life-saving therapies. Nephrologists emphasised that newer binders enabled them to maintain RAASis in patients who otherwise require dose reduction or discontinuation:

*“I do not eliminate the ARB or the beta-blocker. I add the appropriate treatment to compensate. I prefer to adjust and complement rather than suspend.”* (ID02, Female, 60 years)

### Navigating systemic friction and care fragmentation

Despite therapeutic advances, nephrologists highlighted substantial organisational and administrative barriers that constrained consistent delivery of optimal care. Access to newer potassium binders was described as uneven and often delayed by regional prescribing policies and prior authorisation requirements:

*“In some regions, we are obliged to start with Resincalcio before requesting newer binders. The problem is its poor tolerability, which leads to poor adherence and recurrence.”* (ID12, Male, 49 years)

These access constraints were perceived as particularly problematic in outpatient settings, where early intervention could prevent clinical deterioration and avoid hospitalisation.

Participants also described fragmented communication across specialties, particularly between nephrology, emergency medicine, and primary care. Patients presenting with hyperkalaemia in acute settings were often discharged with suspended RAASis and no clear plan for reinitiation:

*“The patient goes to the emergency department, they withdraw all treatment, and nobody restarts it afterwards. When the patient returns months later, they have been without essential medication all that time.”* (ID05, Female, 56 years)

Emerging technologies such as remote potassium monitoring were viewed as promising but inconsistently implemented. Limited integration into routine practice was attributed to organisational limitations and resource constraints.

### The human element: trust, communication, and the role of nursing

Across interviews, nephrologists emphasised that successful hyperkalaemia management depended not only on pharmacological interventions but also on patient understanding, trust, and sustained engagement. Clear communication was described as essential for adherence, particularly in patients managing complex regimens and fluctuating potassium levels:

*“Patients need to understand why they are taking the treatment. Transparency and common language are key to adherence.”* (ID10, Male, 47 years)

Renal nursing staff were consistently identified as central to patient education and empowerment. Nurses were described as trusted intermediaries who reinforced dietary advice, clarified medication instructions, and ensured continuity of care across care settings:

*“If the nurses leave, the unit is paralysed. They are the ones who train the patients and constantly reinforce self-care.”* (ID07, Male, 59 years)

At the same time, clinicians acknowledged structural constraints limiting non-pharmacological counselling, including time pressures, limited access to dietitians, and variability in training.

### The unmeasured burden: when biochemistry overshadows lived experience

Nephrologists were acutely aware that the burden of hyperkalaemia management often extended beyond laboratory values. Strict dietary restrictions, treatment complexity, and pill burden were described as major sources of frustration and social limitation:

*“They tell me they do not know what to eat anymore. They avoid going out because of the diet or the inconvenience of taking the medication.”* (ID12, Male, 49 years)

Newer potassium binders were perceived as improving daily life by reducing gastrointestinal discomfort and the stigma associated with traditional resins, allowing some patients to “recover the pleasure of eating”:

*“Patients who do not tolerate Resincalcio clearly experience a better quality of life with the new drugs.”* (ID11, Female, 42 years)

Despite this awareness, formal assessment of HRQoL was rarely integrated into routine practice. Most clinicians relied on informal clinical impressions rather than validated instruments:

*“We assume more than we measure. We should incorporate more questionnaires and systematic evaluations of quality of life.”* (ID03, Female, 58 years)

This gap between perceived burden and formal measurement highlights an unmet need in routine hyperkalaemia management.

## Discussion

This qualitative study provides in-depth insights into the real-world management of hyperkalaemia among nephrologists in Spain, suggesting a shift towards proactive potassium control aimed at preserving cardiorenal protection. Although clinicians reported strong alignment with international guidelines on maintaining RAASi therapy, they also described systemic and administrative constraints that limited optimal implementation. These findings reflect broader challenges in translating clinical evidence into routine practice within chronic disease management. Because this was a qualitative study based on clinician interviews, findings should be interpreted as perspectives on routine practice rather than as quantitative evidence of clinical effectiveness, cost-effectiveness, or causal relationships.

### Structured care pathway and renin–angiotensin–aldosterone system inhibitor preservation

Nephrologists in this study consistently described a structured, stepwise approach to hyperkalaemia management that prioritises the maintenance of RAASi therapy, aligning with the Kidney Disease: Improving Global Outcomes and European Society of Cardiology guidelines [40]. Participants viewed RAASi discontinuation not as a standard safety measure but as a ‘last resort’ undertaken only when metabolic and dietary interventions had failed. This aligns with consensus that the long-term survival benefits of RAASi therapy outweigh the risks associated with mild-to-moderate hyperkalaemia [41]. This proactive stance contrasts with findings from earlier real-world observational studies, where clinical inertia often prevailed, and hyperkalaemia frequently triggered immediate RAASi discontinuation or down-titration rather than management. By exhausting conservative measures (diet, diuretics, bicarbonate) before down-titrating RAASi, Spanish nephrologists adhere to a “treat the potassium, not the RAASi” philosophy, which is essential for delaying dialysis and improving cardiovascular outcomes in CKD [42,43]. However, because this study did not measure patient outcomes, these interpretations reflect clinicians’ rationale for preserving RAASi rather than demonstrated clinical benefit within the study sample. More broadly, these findings illustrate how clinicians operationalise guideline recommendations in the face of uncertainty, prioritising long-term outcome optimisation over short-term risk avoidance. Such decision-making patterns are relevant across chronic disease areas in which evidence-based therapies carry manageable adverse effects yet are frequently underutilised in routine practice.

### The role of newer binders and patient preference

Participants described newer potassium binders (patiromer and SZC) as transformative factors in clinical practice. In contrast to traditional resins (CPS), which are associated with poor tolerability and high discontinuation rates [44], newer agents were perceived as effective ‘enablers’ of RAASi therapy. These qualitative insights align closely with the quantitative results of the APPETIZE study, which reported a strong patient preference for the palatability and texture of newer binders compared with traditional resins [45]. Participants in this study confirmed that improved tolerability is a key driver of adherence, facilitating a shift from reactive, emergency-based management to proactive outpatient stabilisation. Although adherence and treatment continuity were not quantitatively assessed in this study, participants consistently perceived tolerability and ease of use as important contributors to sustained treatment use. These findings support the notion that enabling therapies, which preserve patient autonomy and reduce treatment burden, may generate greater real-world value than restrictive interventions, even when acquisition costs are higher. From a health economics perspective, this underscores the importance of intervention acceptability as a determinant of real-world effectiveness that is often underrepresented in cost-effectiveness models focused primarily on clinical endpoints.

### Systemic barriers and economic paradoxes

Despite the clinical value of newer binders, our study identified clinician-reported administrative barriers to their use. The requirements for *visado* (administrative pre-authorisation) and step-edit protocols, often mandating prior failure of traditional resins, create a bottleneck that delays optimal care delivery. This creates a paradox in which clinical guidelines recommend early intervention to maintain RAASi, whereas reimbursement policies often delay access until the patients have failed older, less-tolerated therapies.

From a health economics perspective, this administrative burden highlights a potential misalignment between clinical guidelines and reimbursement mechanisms. It is consistent with recent cost-effectiveness models in the Spanish setting, which suggest that the acquisition costs of newer binders may be offset by savings associated with delayed CKD progression and reduced heart failure hospitalisations [46]. Importantly, although newer binders may be cost-effective at the health system level, they are not necessarily cost saving within short-term pharmaceutical budgets, particularly when downstream savings accrue outside nephrology or beyond annual budget cycles. Our findings suggest that, from clinicians’ perspectives, current access hurdles may prioritise short-term pharmaceutical budget protection over long-term system efficiency. Moreover, this tension reflects a broader gap between economic evidence generation and clinical implementation, whereby clinicians focus on individual patient benefit, whereas payers must manage population-level affordability under uncertainty. Future research should formally evaluate whether the access barriers identified in this qualitative study translate into measurable differences in RAASi persistence, healthcare utilisation, patient outcomes, and costs.

### Patient engagement and the burden of care

Adherence was strongly linked to “knowledge activation”; however, the psychosocial burden of dietary restrictions remains a significant and often unmeasured challenge. From a health economics perspective, these findings underscore the importance of intervention acceptability as a key determinant of real-world effectiveness. The theme of social isolation reported by our participants mirrors recent findings in broader CKD populations, in which strict low-potassium diets have been shown to impair social participation and contribute to a diminished sense of self [47].

While nephrologists prioritise biochemical control, this qualitative evidence suggests a gap in routine care: the trade-off between dietary freedom and pill burden is rarely formally assessed using patient-reported outcome measures (PROMs). This points to an opportunity for future research and policy to integrate PROMs more systematically into clinical practice and economic evaluations, thereby capturing the full burden of treatment and informing patient-centred decision-making. As HRQoL was discussed from the perspective of clinicians rather than measured directly from patients, future studies should incorporate validated patient-reported outcome instruments to quantify the impact of hyperkalaemia management strategies on patients’ daily functioning, treatment burden, and well-being.

## Limitations

While this study provides valuable insights, it has some limitations worth noting. First, the findings reflect the perspectives of a relatively small group of practising nephrologists and may not capture the full diversity of clinical experiences across Spain. Second, there is potential selection bias in relation to practice setting; as the majority of participants (83%) practised in university- or tertiary-level hospitals, the findings may not fully capture the challenges faced by nephrologists in smaller community hospitals with fewer resources. Third, purposive sampling of clinicians with experience prescribing newer binders may have introduced an ‘early adopter’ bias, with participants potentially holding more favourable or informed views than clinicians less familiar with these therapies. Finally, because the study relied solely on clinician self-report, we were unable to directly assess patient adherence or the administrative rationale of payers regarding *visado* restrictions. Similarly, the study did not collect patient-reported outcome data, prescribing records, healthcare utilisation data, or cost data; therefore, conclusions regarding HRQoL, RAASi persistence, economic value, or policy impact should be interpreted as hypotheses generated from clinician perspectives rather than as directly measured outcomes. Future research incorporating PROMs and payer perspectives would provide a more comprehensive view of hyperkalaemia management.

### Implications for policy and research

This study highlights that nephrologists in Spain prioritise RAASi preservation in hyperkalaemia management and view newer potassium binders as important tools to support this goal. However, clinicians perceived that implementation of this strategy is often constrained by administrative access barriers, such as *visado* requirements, and by fragmented care pathways across specialties. From a policy perspective, these findings indicate potential barriers to the implementation of guideline-recommended, cardiorenal-protective care. However, because this was a qualitative study and did not evaluate reimbursement decisions, budget impact, or patient-level outcomes directly, these findings should be interpreted as areas for further investigation rather than evidence of policy inefficiency. Newer agents have improved treatment tolerability; however, the ongoing burden of dietary restrictions and the limited use of formal HRQoL assessment tools point to persistent gaps in patient-centred care. To fully realise the cardiorenal benefits of RAASi therapy, healthcare policies should prioritise streamlined access to effective potassium management strategies and support the integration of patient-reported outcomes into routine clinical decision-making.

From a research perspective, future studies are needed to bridge the gap between reported clinician intent and actual prescribing behaviour. Large-scale, real-world registry studies could help quantify the impact of administrative barriers on RAASi persistence and downstream clinical outcomes. Additionally, given the burden associated with dietary restrictions identified in this study, further research should prioritise the development and validation of PROMs that capture quality-of-life trade-offs between dietary freedom and medication burden, to better inform clinical practice and health-economic evaluations. Future health economic studies should also examine whether earlier or less administratively burdensome access to newer potassium binders is associated with improved RAASi persistence, reduced acute-care use, delayed CKD progression, and acceptable value for money from the perspective of the Spanish National Health System.

## Supporting information

S1 File Semi-structured interview guide. (DOCX)

S2 Table Codebook and code frequencies. (DOCX)

S3 Table Anonymised participant characteristics. (DOCX)

S4 Table Additional anonymised illustrative quotations organised by theme. (DOCX)
