# Supplementary material for: Navigating the potassium dilemma: a qualitative study of nephrologists’ strategies for renin–angiotensin–aldosterone system inhibitor preservation and hyperkalaemia management in Spain
Source: PLoS One. 2026 Jul 30;21(7):e0354854. doi: 10.1371/journal.pone.0354854 (PMC13422876; doi:10.1371/journal.pone.0354854)
Supplement: S1 File — (DOCX) [file pone.0354854.s001.docx]

**S1 File. Semi-structured interview guide**

Note: Interviews were conducted in Spanish. The semi-structured interview guide presented below is an English translation of the Spanish-language guide used during data collection. The guide was used flexibly, with follow-up probes adapted to participants’ responses.

Study title: Navigating the potassium dilemma: a qualitative study of nephrologists’ strategies for renin–angiotensin–aldosterone system inhibitor preservation and hyperkalaemia management in Spain

**Part I. Introduction and professional context**

1. **Introduction**

Thank you for participating in this study. To begin, could you please briefly introduce yourself and describe your current role as a nephrologist?

Potential probes:

- Current clinical role and practice setting
- Main patient populations seen in routine practice
- Experience managing patients with chronic kidney disease and hyperkalaemia

**Part II. Hyperkalaemia management options**

1. **General approach to hyperkalaemia management**

Could you describe your general approach to managing patients with recurrent hyperkalaemia?

Potential probes:

- First-line strategies when high serum potassium is identified in patients with chronic kidney disease
- Differences between management in earlier versus later stages of chronic kidney disease
- Factors that influence treatment escalation or de-escalation

1. **Views on available treatment options**

What are your views on the current treatment options available for managing hyperkalaemia in patients with chronic kidney disease?

Potential probes:

- Dietary modification
- Traditional potassium binders, such as calcium polystyrene sulfonate
- Newer potassium binders, including patiromer and sodium zirconium cyclosilicate
- Diuretics
- Adjustment of renin–angiotensin–aldosterone system inhibitor therapy
- Bicarbonate therapy
- Sodium-glucose cotransporter-2 inhibitors

1. **Important treatment attributes**

What treatment attributes are most important to you when choosing a management strategy for patients with chronic or recurrent hyperkalaemia?

Potential probes:

- Potassium-lowering effectiveness
- Speed of onset
- Tolerability and adverse effects
- Ease of administration
- Treatment burden and patient adherence
- Access, reimbursement, and prescribing restrictions
- Patient preferences

Why are these attributes important in your clinical decision-making?

**Part III. Hyperkalaemia symptoms, detection, and diagnosis**

1. **Timing and detection of hyperkalaemia**

At what stage of chronic kidney disease do you most commonly observe hyperkalaemia in your practice?

Potential probes:

- Whether hyperkalaemia is usually detected through routine laboratory monitoring, symptoms, or acute events
- Differences between acute and chronic hyperkalaemia
- Factors that increase the likelihood of recurrent hyperkalaemia

1. **Diagnostic procedures and monitoring**

What tests or assessments are most commonly used to diagnose and monitor hyperkalaemia in your practice?

Potential probes:

- Serum potassium monitoring
- Electrocardiogram assessment
- Blood gas analysis
- Monitoring frequency in stable versus unstable patients
- Differences in assessment between acute and chronic hyperkalaemia

**Part IV. Clinical role, treatment decisions, and patient communication**

1. **Role in prescribing and care coordination**

Are you usually responsible for prescribing treatments for patients with chronic kidney disease and hyperkalaemia?

Potential probes:

- Role of nephrologists in initiating or adjusting treatment
- Involvement of other specialists, such as cardiologists, internists, emergency physicians, or primary care physicians
- Communication and coordination across specialties
- Reinitiation of renin–angiotensin–aldosterone system inhibitor therapy after acute hyperkalaemia episodes

1. **Medication practices and RAASi management**

Which treatments for chronic kidney disease most often contribute to hyperkalaemia risk in your experience?

Potential probes:

- Renin–angiotensin–aldosterone system inhibitors
- Mineralocorticoid receptor antagonists
- Other relevant medications or comorbidities
- Balancing potassium control with preservation of cardiorenal protective therapies
- Circumstances in which RAASi dose reduction or discontinuation is considered

1. **Treatment selection and sequencing**

Which treatments do you prescribe or recommend for hyperkalaemia, and how do you sequence them?

Potential probes:

- Differences according to comorbidities or chronic kidney disease stage
- Use of traditional versus newer potassium binders
- Considerations when choosing sodium-based versus non-sodium-based treatments
- Efficacy, tolerability, familiarity, patient preference, cost, access, and reimbursement
- Regional or institutional prescribing restrictions

1. **Patient communication and education**

How do you explain hyperkalaemia management decisions to patients?

Potential probes:

- How treatment rationale is communicated
- How risks and benefits are explained
- Strategies used to support adherence
- Role of nurses, dietitians, or other healthcare professionals in patient education
- Use of written materials, digital tools, or patient support resources

1. **Quality of life and treatment burden**

How would you describe the quality of life of patients living with acute or chronic hyperkalaemia?

Potential probes:

- Impact of dietary restrictions
- Impact of medication burden
- Impact on daily routines, emotional well-being, and social life
- Differences between traditional and newer potassium binders from the patient perspective
- Whether health-related quality of life is formally assessed in routine practice
- Whether patient-reported outcome measures should be integrated more systematically into clinical care

**Part V. Patient support and care pathway improvement**

1. **Patient support programmes and resources**

Are there any support programmes, educational materials, or resources that you recommend to patients with hyperkalaemia?

Potential probes:

- Dietary education
- Nursing-led education
- Digital tools or applications
- Multidisciplinary support

1. **Opportunities to improve the care pathway**

If you could change anything about the care pathway for patients with hyperkalaemia, what would it be?

Potential probes:

- Access to treatments
- Prescribing or authorisation processes
- Multidisciplinary coordination
- Monitoring and follow-up
- Patient education and support
- Integration of quality-of-life assessment

**Closing question**

Before we close, is there anything else about the management of hyperkalaemia in patients with chronic kidney disease that we have not discussed and that you consider important?

Thank you for your participation.
