# Supplementary material for: Navigating the potassium dilemma: a qualitative study of nephrologists’ strategies for renin–angiotensin–aldosterone system inhibitor preservation and hyperkalaemia management in Spain
Source: PLoS One. 2026 Jul 30;21(7):e0354854. doi: 10.1371/journal.pone.0354854 (PMC13422876; doi:10.1371/journal.pone.0354854)
Supplement: S2 Table — (DOCX) [file pone.0354854.s002.docx]

**S1 Table. Codebook and code frequencies**

| **Theme, sub-theme, code** | **Frequency  (total mentions)** | **Participants (N=12)** |
| --- | --- | --- |
| **Theme 1: Safeguarding cardiorenal protection through stepwise clinical reasoning** |  |  |
| *Staged management beginning with dietary and metabolic correction* |  |  |
| - Acute vs chronic hyperkalemia management | 54 | 12/12 |
| - Early identification of hyperkalaemia | 27 | 11/12 |
| - Routine laboratory monitoring | 27 | 12/12 |
| - Use of electrocardiogram and blood gas analysis | 23 | 11/12 |
| - Dietary counselling and potassium restriction | 32 | 12/12 |
| - Fluid and potassium removal strategies (diuretics, residual diuresis, dialysis efficiency) | 24 | 12/12 |
| - Use of bicarbonate in metabolic acidosis | 31 | 12/12 |
| - Use of sodium-glucose cotransporter-2 inhibitors and their role in potassium balance | 14 | 12/12 |
| *Maintaining renin–angiotensin–aldosterone system inhibitors as a primary clinical imperative* |  |  |
| - Therapeutic decision-making by comorbidity or treatment algorithm | 46 | 12/12 |
| *Shifting toward individualised and evidence-based dietary practice* |  |  |
| - Reflections on outdated dietary practices | 9 | 6/12 |
| **Theme 2: A therapeutic shift: new binders as enablers of care** |  |  |
| *Modern binders viewed as a fundamental shift in outpatient management* |  |  |
| - Use of new binders | 92 | 12/12 |
| *Superior tolerability and usability compared with traditional resins* |  |  |
| - Use of traditional binders | 50 | 12/12 |
| - Adherence barriers (taste, texture, side effects) | 57 | 12/12 |
| - Ease of use and dosing frequency | 22 | 11/12 |
| - Switching between treatments due to intolerance | 19 | 10/12 |
| *Facilitating the continuity of life-saving therapies in predialysis chronic kidney disease* |  |  |
| - Adjustment of renin–angiotensin–aldosterone system inhibitors therapy | 64 | 12/12 |
| **Theme 3: Navigating system friction and care fragmentation** |  |  |
| *Navigating inconsistent access and administrative hurdles to treatment* |  |  |
| - Restricted and inconsistent access to new binders | 56 | 12/12 |
| - Regional differences in formularies or hospital protocols | 14 | 8/12 |
| *Overcoming fragmented coordination across medical specialties* |  |  |
| - Interaction between nephrology, cardiology, and primary care | 38 | 11/12 |
| *Addressing the slow adoption of remote monitoring and digital innovations* |  |  |
| - Emerging technologies and future perspectives (home testing) | 22 | 11/12 |
| **Theme 4: The human element: trust, communication, and the role of nursing** |  |  |
| *Fostering treatment adherence through transparent clinician communication* |  |  |
| - Patient communication about treatment rationale | 30 | 8/12 |
| - Perceived risk-benefit balance | 27 | 12/12 |
| *The essential role of renal nursing in patient education and empowerment* |  |  |
| - Role of specialized nursing in patient education | 23 | 11/12 |
| - Use of educational tools and dietary apps | 22 | 11/12 |
| - Patient trust and long-term relationship with nephrologist | 20 | 10/10 |
| *Identifying structural constraints in the delivery of comprehensive nutritional guidance* |  |  |
| Need for better nutritional education among professionals | 8 | 6/12 |
| **Theme 5: The unmeasured burden: when biochemistry overshadows lived experience** |  |  |
| *Understanding how hyperkalaemia management restricts social and daily participation* |  |  |
| - Impact of dietary restrictions on emotional well-being | 6 | 5/12 |
| - Impact of treatment on daily routine and social life | 13 | 10/12 |
| *Recognising the restoration of well-being through improved pharmacological tolerance* |  |  |
| - Perceived quality of life improvement with new binders | 34 | 10/12 |
| *The challenge of relying on clinical intuition over formal quality-of-life assessment* |  |  |
| - Use of structured health-related quality-of-life questionnaires | 14 | 8/12 |

**Note**: Main themes are presented in bold; sub-themes are indicated in italics. **Definitions**: Frequency refers to the total number of times the code was identified across the entire dataset of 12 transcripts. Participants indicate the number of unique nephrologists (out of 12) who discussed the concept.
