# Supplementary material for: Navigating the potassium dilemma: a qualitative study of nephrologists’ strategies for renin–angiotensin–aldosterone system inhibitor preservation and hyperkalaemia management in Spain
Source: PLoS One. 2026 Jul 30;21(7):e0354854. doi: 10.1371/journal.pone.0354854 (PMC13422876; doi:10.1371/journal.pone.0354854)
Supplement: S3 Table — (DOCX) [file pone.0354854.s003.docx]

**S3 Table. Aggregated participant characteristics**

This supplementary table presents de-identified screening characteristics for the 12 nephrologists who participated in the study. Names, locations, institution names, interview dates, meeting links, consent-administration fields and other logistical details have been removed. Exact ages, exact years in clinical practice and direct patient-care percentages have been banded to support anonymisation.

| **Participant ID** | **Age group (years)** | **Gender** | **Years in clinical practice after residency** | **Primary practice setting** | **Direct patient care time** |
| --- | --- | --- | --- | --- | --- |
| **ID01** | **50-59** | **Male** | **16-25** | **University / tertiary hospital** | **≥75%** |
| **ID02** | **≥60** | **Female** | **>25** | **University / tertiary hospital** | **50-74%** |
| **ID03** | **50-59** | **Female** | **16-25** | **University / tertiary hospital** | **≥75%** |
| **ID04** | **40-49** | **Male** | **16-25** | **General hospital** | **≥75%** |
| **ID05** | **50-59** | **Female** | **16-25** | **University / tertiary hospital** | **≥75%** |
| **ID06** | **50-59** | **Male** | **16-25** | **University / tertiary hospital** | **≥75%** |
| **ID07** | **50-59** | **Male** | **16-25** | **University / tertiary hospital** | **≥75%** |
| **ID08** | **40-49** | **Female** | **10-15** | **University / tertiary hospital** | **≥75%** |
| **ID09** | **≥60** | **Male** | **>25** | **General hospital** | **≥75%** |
| **ID10** | **40-49** | **Male** | **16-25** | **University / tertiary hospital** | **≥75%** |
| **ID11** | **40-49** | **Female** | **10-15** | **University / tertiary hospital** | **≥75%** |
| **ID12** | **40-49** | **Male** | **16-25** | **University / tertiary hospital** | **≥75%** |

Notes: All participants were practising nephrologists in Spain, had direct responsibility for CKD management, were involved in treatment decisions for recurrent hyperkalaemia, and were aware of both Veltassa (patiromer) and Lokelma (sodium zirconium cyclosilicate).

Abbreviation: CKD, chronic kidney disease.
