# Supplementary material for: Navigating the potassium dilemma: a qualitative study of nephrologists’ strategies for renin–angiotensin–aldosterone system inhibitor preservation and hyperkalaemia management in Spain
Source: PLoS One. 2026 Jul 30;21(7):e0354854. doi: 10.1371/journal.pone.0354854 (PMC13422876; doi:10.1371/journal.pone.0354854)
Supplement: S4 Table — (DOCX) [file pone.0354854.s004.docx]

**S4 Table. Detailed quotations by theme**

| **Theme** | **Sub-theme** | **Participant ID** | **Additional anonymised quotation (English translation)** |
| --- | --- | --- | --- |
| Theme 1. Safeguarding cardiorenal protection through stepwise clinical reasoning | Staged management beginning with dietary and metabolic correction | ID01 | In patients with stage 4 or 5 chronic kidney disease who are not yet on dialysis, the main approach is dietary advice, optimisation of metabolic acidosis when present, and potassium binders when needed. In patients on dialysis, there is no residual renal route to remove potassium, so binders are used more directly. |
| Theme 1. Safeguarding cardiorenal protection through stepwise clinical reasoning | Staged management beginning with dietary and metabolic correction | ID07 | In acute hyperkalaemia, the priority is to stabilise the patient and lower potassium rapidly. In chronic hyperkalaemia, the strategy is different: diet comes first, followed by correction of acidosis and potassium binders when potassium remains high. |
| Theme 1. Safeguarding cardiorenal protection through stepwise clinical reasoning | Maintaining RAASi as a primary clinical imperative | ID01 | If we reduce or stop ACE inhibitors or angiotensin receptor blockers, we may lower potassium, but we are also removing nephroprotective and cardioprotective drugs. In the medium and long term, that can increase morbidity and mortality. |
| Theme 1. Safeguarding cardiorenal protection through stepwise clinical reasoning | Maintaining RAASi as a primary clinical imperative | ID02 | I do not remove the angiotensin receptor blocker or the beta-blocker. I add the appropriate treatment to compensate for the potassium effect. I prefer to adjust and complement treatment before suspending it. |
| Theme 1. Safeguarding cardiorenal protection through stepwise clinical reasoning | Shifting toward individualised and evidence-based dietary practice | ID06 | Traditionally, we insisted a lot on limiting fruit, vegetables and nuts. However, we now try not to be so strict because an overly restrictive diet can be counterproductive and deprive patients of important nutrients. |
| Theme 2. A therapeutic shift: new binders as enablers of care | Modern binders viewed as a fundamental shift in outpatient management | ID01 | The newer binders have given us a therapeutic arsenal that prevents many urgent situations. Years ago, I sometimes had to place an emergency catheter at three in the morning for a patient with very high potassium. That has not happened to me in recent years. |
| Theme 2. A therapeutic shift: new binders as enablers of care | Superior tolerability and usability compared with traditional resins | ID01 | Traditional resin is effective, but it is very poorly tolerated and many patients simply do not take it. They collect it from the pharmacy and keep it at home, but it never actually passes their mouth. |
| Theme 2. A therapeutic shift: new binders as enablers of care | Superior tolerability and usability compared with traditional resins | ID06 | Traditional binders are increasingly used less because patients tolerate them very badly. Many describe them as like eating plaster, they cause constipation, and the experience is unpleasant, so adherence is low. |
| Theme 2. A therapeutic shift: new binders as enablers of care | Facilitating continuity of life-saving therapies in predialysis CKD | ID03 | There are situations, particularly in nephro-cardiology clinics, where these medicines are necessary and cannot be withdrawn. When hyperkalaemia appears because of them, the option is to add a potassium binder. |
| Theme 2. A therapeutic shift: new binders as enablers of care | Individualising binder choice according to clinical profile | ID11 | For me, the main comorbidity that influences the choice between Lokelma and Veltassa is heart failure, because of the risk of sodium retention associated with Lokelma. That is the key clinical factor when choosing between them. |
| Theme 3. Navigating systemic friction and care fragmentation | Navigating inconsistent access and administrative hurdles to treatment | ID01 | The newer binders are funded, but there are administrative restrictions. In my region, they are only authorised if the patient has previously tried traditional resin and not tolerated it. |
| Theme 3. Navigating systemic friction and care fragmentation | Navigating inconsistent access and administrative hurdles to treatment | ID06 | In theory, Spanish guidance says that the patient must first try traditional resin and only move to a newer binder if it is not tolerated. In practice, this visado process is cumbersome and can delay treatment when you already know the patient would benefit. |
| Theme 3. Navigating systemic friction and care fragmentation | Navigating inconsistent access and administrative hurdles to treatment | ID02 | If I could change one thing in the care pathway, I would remove mandatory authorisation for the newer medicines, or at least reform it so that decisions are made by health professionals rather than by an administrative office. |
| Theme 3. Navigating systemic friction and care fragmentation | Overcoming fragmented coordination across medical specialties | ID05 | Many times the problem is the lack of connection between specialties. A patient goes to the emergency department with acute hyperkalaemia, all treatment is stopped there, and afterwards no one realises that the patient has remained without essential medication. |
| Theme 3. Navigating systemic friction and care fragmentation | Addressing the slow adoption of remote monitoring and digital innovations | ID10 | It would be very valuable to measure potassium at home, in a similar way to how patients with diabetes check glucose. The technology exists, but it still needs validation and integration into routine clinical practice. |
| Theme 4. The human element: trust, communication, and the role of nursing | Fostering treatment adherence through transparent clinician communication | ID02 | I always try to speak to patients with complete clarity. I explain that there are different medicines, with different mechanisms of action and possible adverse effects, and I tell them the truth while also explaining that not every listed adverse effect will necessarily occur. |
| Theme 4. The human element: trust, communication, and the role of nursing | Fostering treatment adherence through transparent clinician communication | ID08 | It is difficult for patients to understand the importance of controlling hyperkalaemia because it is often asymptomatic. They do not feel anything, so they do not perceive the risk that high potassium can cause serious cardiac problems. |
| Theme 4. The human element: trust, communication, and the role of nursing | The essential role of renal nursing in patient education and empowerment | ID01 | Who has the greatest influence on the patient? The person who spends the most time with them. In dialysis, the patient spends hours with the nurse several days each week. Nursing has to lead patient empowerment, medication-taking and diet education. |
| Theme 4. The human element: trust, communication, and the role of nursing | The essential role of renal nursing in patient education and empowerment | ID07 | The nephrologist could be away from the unit for a couple of months and everything would continue, but if the nurses leave, the unit stops. They train patients, explain the process and constantly reinforce self-care. |
| Theme 4. The human element: trust, communication, and the role of nursing | Structural constraints in comprehensive nutritional guidance | ID12 | There is still a lack of training in nutritional management for renal patients, especially around hyperkalaemia. Some professionals continue to apply overly restrictive dietary recommendations, which can lead to malnutrition, constipation and, paradoxically, more hyperkalaemia. |
| Theme 5. The unmeasured burden: when biochemistry overshadows lived experience | How hyperkalaemia management restricts social and daily participation | ID12 | Patients often tell me they no longer know what to eat, that they cannot go out with friends or enjoy a meal outside the home. Carrying and preparing the sachet in front of others is uncomfortable, so some prefer to stay at home. |
| Theme 5. The unmeasured burden: when biochemistry overshadows lived experience | How hyperkalaemia management restricts social and daily participation | ID06 | Quality of life is affected above all by the restrictions we impose, rather than by hyperkalaemia itself. Patients are told to limit protein, to be careful with fruit and vegetables, and if they also have diabetes, to control carbohydrates. In the end, everything becomes a restriction. |
| Theme 5. The unmeasured burden: when biochemistry overshadows lived experience | Restoration of well-being through improved pharmacological tolerance | ID12 | With the newer binders, patients notice a major difference. They tolerate them much better, nausea and constipation improve, and they are less embarrassed about taking the treatment. |
| Theme 5. The unmeasured burden: when biochemistry overshadows lived experience | Restoration of well-being through improved pharmacological tolerance | ID01 | Even with the newer binders, there is still an impact on quality of life because I am adding another medication to patients who may already take 10, 12 or 14 medicines a day. |
| Theme 5. The unmeasured burden: when biochemistry overshadows lived experience | Relying on clinical intuition over formal quality-of-life assessment | ID03 | We do few quality-of-life tests, and we should do more. Many times we assume that treatment improves the patient’s quality of life, but we do not always verify it. Today, we assume more than we measure. |
| Theme 5. The unmeasured burden: when biochemistry overshadows lived experience | Relying on clinical intuition over formal quality-of-life assessment | ID04 | I had never specifically considered assessing quality of life related to hyperkalaemia. I have used questionnaires for chronic kidney disease in general, but not validated instruments that address the impact of hyperkalaemia or its treatment specifically. |
